# The Environmental Impact of an Italian-Mediterranean Dietary Pattern Based on the EAT-Lancet Reference Diet (EAT-IT)

**DOI:** 10.3390/foods11213352

**Published:** 2022-10-25

**Authors:** Massimiliano Tucci, Daniela Martini, Mirko Marino, Cristian Del Bo’, Valentina Vinelli, Paola Biscotti, Carlotta Parisi, Ramona De Amicis, Alberto Battezzati, Simona Bertoli, Marisa Porrini, Patrizia Riso

**Affiliations:** 1 Department of Food, Environmental and Nutritional Sciences (DeFENS), Università degli Studi di Milano, 20133 Milan, Italy; 2 International Center for the Assessment of Nutritional Status (ICANS), Università degli Studi di Milano, 20133 Milan, Italy

**Keywords:** sustainable healthy diet, planetary healthy diet, nutrition, sustainability, environmental impact, Mediterranean diet, dietary guidelines

## Abstract

The definition of a healthy and sustainable diet is nowadays considered pivotal, but data related to environmental outcomes are still debated. In this study, we compared the carbon (CF) and water footprints (WF) of an Italian-Mediterranean (EAT-IT) dietary pattern designed on the “Planetary diet”, with a pattern based on the Italian Dietary Guidelines (IDG). The influence of different food categories and food choices on environmental impact was assessed. To this aim, weekly dietary patterns were developed, considering food categories and related portions and frequencies of consumption. Results show that the EAT-IT dietary pattern, compared to the IDG, had a significantly lower CF (2.82 ± 1.07 and 3.74 ± 0.92 kg CO_2_/day, respectively) but not WF. Protein-rich foods were the main contributors to CF and WF in both dietary patterns. The increased substitution of frozen instead of fresh foods, imported instead of local fruits, greenhouse-grown instead of seasonal vegetables, and processed legume-based foods instead of unprocessed legumes caused an increasing worsening of the CF in both patterns, but with different magnitudes. Our analysis indicated that the EAT-IT dietary pattern can be considered sustainable for CF, but individual choices are likely to largely affect the final environmental outcomes.

## 1. Introduction

It is well known that diet is crucial for the promotion of human health. In 2017, 11 million deaths and 255 million disability-adjusted life-years were estimated to be attributable to dietary risk factors and in particular to high sodium intake and low consumption of whole grains, fruit and vegetables, and nuts [1].

More recently, greater consideration is being given to the impact that diet and the entire food system can exert on the health of the planet, depending on how actual eating behavior and food choices affect production. Indeed, the food system has been shown to account for over a third of global human-caused greenhouse gas emissions [2,3], half of the land use and over two-thirds of freshwater use [4,5]. For this reason, the food system is considered one of the principal drivers of climate change, which in turn impacts foods through decreasing food quantity, biodiversity, and nutrient content [6,7].

It is therefore quite clear that there is a challenge to develop and provide to the global population, which is constantly growing, diets that are healthy, with a low environmental impact but, at the same time, are socio-culturally acceptable and economically accessible for all [8,9]. In this scenario, a global modeling analysis was applied to the evaluation of the different dietary patterns identified for more than 150 countries, combining nutrient levels, diet-related and weight-related chronic disease mortality, and environmental impacts. Overall, the results obtained demonstrated that plant-based dietary patterns, aligned with the current evidence on healthy eating, can generally lead to a simultaneous reduction of both health and environmental impacts in all regions, despite differences among countries in terms of resource use [10].

With the intention to develop a global healthy and sustainable diet (i.e., “win-win” approach), the EAT-Lancet Commission developed a “planetary” healthy reference diet for an intake of 2500 kcal/day [11] characterized by a high consumption of whole grains, vegetables, legumes, nuts, unsaturated oils, with low amounts of seafood and poultry, and low or no added sugar, refined grains, or starchy vegetables and red meat, particularly processed meats which generally have the highest environmental impact. The impact of food and diets on the environment can be calculated thanks to the use of different indicators [12]. However, only few of them have standardized methodologies, among which are the carbon footprint (CF) and the water footprint (WF) [13]. 

This global reference diet aims to confer both improved health and environmental benefits thanks to the definition of planetary boundaries (e.g., cropland use, biodiversity loss, water use, greenhouse-gas emissions, and nitrogen and phosphorus pollution) which will allow a reduction of environmental degradation caused by food production. The shift from the current global diet to this planetary diet has been estimated to be able to prevent approximately 11 million deaths per year and aims to provide healthy diets for a global population of about 10 billion people by 2050 while remaining within a safe operating space for the food system [11,14].

The “Planetary health diet” should be adapted in different countries, taking into consideration food cultures and traditions, and also to improve adherence to the dietary pattern. With this intention, researchers have developed applications of the Planetary health diet in different contexts [15] or have evaluated how current dietary habits differ from this reference diet [16,17].

We have recently developed a Mediterranean dietary pattern in line with the EAT-Lancet Commission reference diet (EAT-IT), based on 2500 kcal/day and adapted to Italian food habits, as published elsewhere [18]. Briefly, this adaptation was made by translating the EAT-Lancet recommendations into a more practical dietary scheme, allocating the different portion sizes and frequencies of consumption within different meals according to Italian dietary habits. The nutritional adequacy of this dietary pattern was compared to the current Italian Dietary Guidelines (IDG) [18], showing that the EAT-IT dietary pattern was higher in nuts and legumes compared to the one based on the IDG. Some potential critical issues emerged, mainly related to the low levels of calcium in the EAT-IT dietary plan compared to the IDG-based one.

The aims of the present study are to (i) estimate the environmental impact of the EAT-IT dietary plan in comparison with the IDG-based one; (ii) elucidate which are the main food categories contributing to the environmental impact of the two dietary plans; and (iii) estimate the variation of the environmental impact of the diet when different types of foods are selected.

## 2. Materials and Methods

### 2.1. Environmental Impact of Foods

The environmental impact of the foods included in the dietary patterns was calculated by using a recently developed multilevel carbon and water footprint dataset of food commodities which assigns footprint values with related uncertainties to food items, starting from peer-reviewed articles and grey literature [13]. Data related to carbon footprint (CF, expressed as kg CO_2_ equivalents/kg or L of product) and water footprint (WF, expressed as L of water/kg or L of product), accounting for greenhouse gas emissions and the consumption of water resources, respectively, were retrieved from the dataset.

Only food items showing both WF and CF values were extracted from the dataset and specifically selected to be used in the dietary plans, as described below.

### 2.2. Development of Dietary Pattern and Dietary Plan 

The methodology used for developing the EAT-IT dietary pattern and the mid/long-term dietary plan based on the EAT-IT dietary pattern have been described elsewhere [18].

Briefly, we firstly defined the EAT-IT-based dietary pattern considering the daily intake provided in the Planetary healthy diet for the eight different food categories defined within their report (i.e., whole grains, tubers or starchy vegetables, vegetables, fruits, dairy foods, protein sources, added fats, and added sugars) [11] and which were converted to weekly amounts (grams/week). These amounts of foods were then allocated into different meals considering the main characteristics of the Mediterranean diet (e.g., sweet breakfast, cereal-based products, fruits, and vegetables at every main meal) and providing alternatives for the different meals in order to allow the long-term feasibility of this dietary pattern. 

Once the dietary pattern was defined, a dietary plan of 2500 kcal/day was developed with recipes and dishes based on food habits of the Italian population, and including five meals per day (breakfast, lunch, dinner, and mid-morning and mid-afternoon snacks).

**Figure 1:**
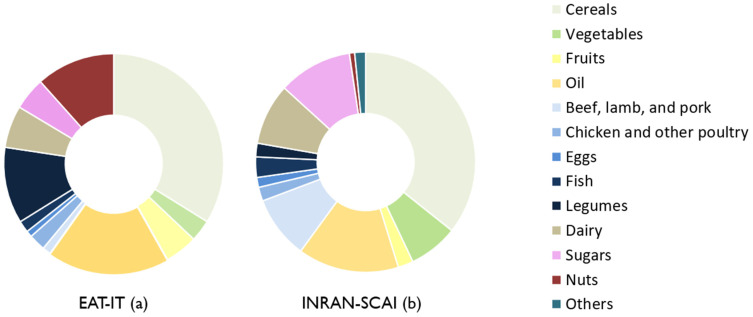
Contribution of different food categories within the EAT-IT dietary pattern (**a**) and habitual Italian diet, based on INRAN-SCAI 2005-2006 data (**b**). Data are reported as mean daily intake percentage of total energy. Legend: EAT-IT: Italian-Mediterranean Dietary Pattern Developed Based on the EAT-Lancet Reference Diet; INRAN-SCAI: Italian National Food Consumption Survey [19].

Similarly to the dietary plan based on EAT-IT, a dietary plan based on the IDG was also developed, following the same procedure described above [18]. A comparison between the contribution of the different food groups in the EAT-IT dietary pattern and in current Italian dietary habits, based on the Italian National Food Consumption Survey INRAN-SCAI 2005–06 data [19], is reported in Figure 1. As shown, the EAT-IT dietary pattern is characterized by a higher contribution of nuts, legumes and a lower contribution of white meat, dairy, and sugars compared to the current Italian dietary intake.

### 2.3. Assessment of the Environmental Impact of the Dietary Patterns

In order to calculate an average daily environmental impact, foods (within the same category) were allocated to the different days of the week, respecting the portions and frequencies of consumption of foods in the two dietary patterns. For this purpose, the RANDOM function of Excel was used. This function allows the generation of a random number sequence, that was then associated to the different food items for their random allocation. The mean daily CF (kg CO_2_ eq./day) and WF (L/day), were calculated for each food category and for the whole dietary patterns.

### 2.4. Analysis of Scenarios Depending on Food Choices

To investigate the impact of different food choices on CF and WF, four different case-studies were selected: (i) use of frozen instead of fresh foods (i.e., bread, fish products, and beans); (ii) use of imported instead of local fruits; (iii) use of greenhouse-grown instead of seasonal vegetables; and (iv) use of processed instead of unprocessed legume-based foods.

For each scenario, increasing levels of substitution (from 25% to 100%) were simulated in order to investigate the impact of increasing use of these products on CF and WF compared to the EAT-IT and IDG dietary patterns in which 0% use of these products was assumed. 

### 2.5. Statistical Analysis 

Data on CF and WF are expressed as mean ± standard deviation, except for variations in the different scenarios that are expressed as delta percentages compared to the EAT-IT and IDG dietary patterns described above.

Differences in the total CF and WF and in the relative footprints from the different food categories were analyzed by using the *t*-test. Statistical analysis was performed by using Student’s *t*-test. The significance level was fixed at *p* < 0.05.

## 3. Results

### 3.1. Environmental Impact of the Two Dietary Patterns

#### 3.1.1. Carbon Footprint

**Figure 2:**
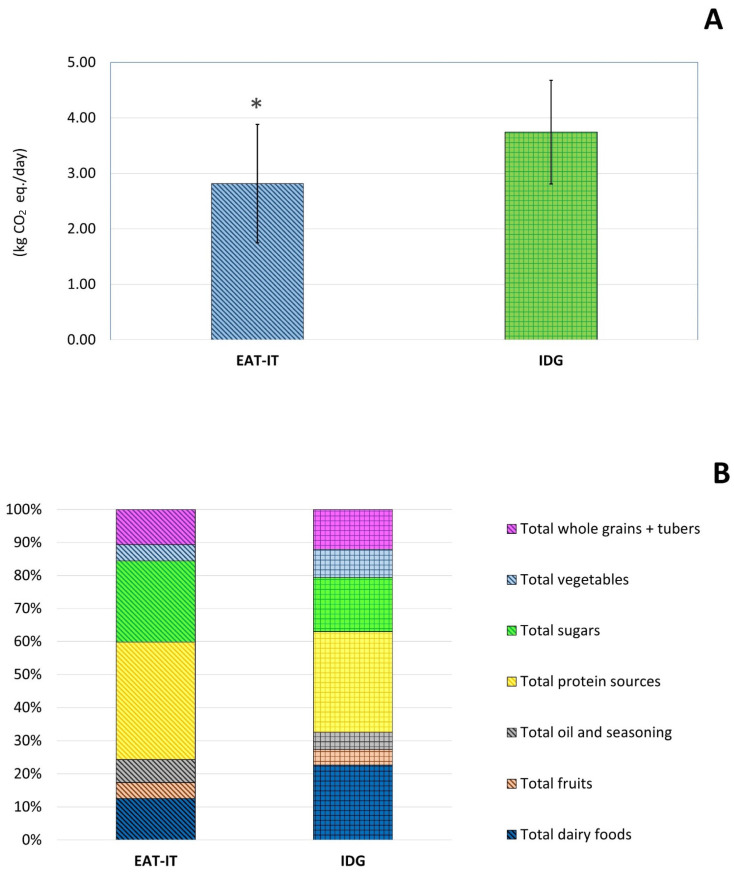

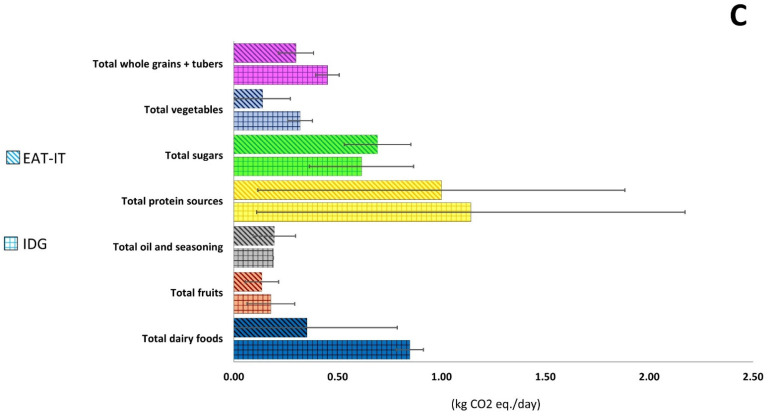
Total (**A**) carbon footprint associated with EAT-IT and IDG dietary patterns and relative (**B**) and percentage (**C**) contribution of the different food groups. Legend: EAT-IT: EAT-Lancet Commission reference diet (adapted to the Italian food habits); IDG: Italian Dietary Guidelines; CF: carbon footprint; *: statistically significant differences (*p* < 0.05).

The environmental impact of the EAT-IT and IDG-based dietary patterns in terms of CF is reported in Figure 2. In detail, Figure 2A shows significantly lower CF for EAT-IT compared to the IDG-based dietary pattern (2.82 ± 1.07 and 3.74 ± 0.92 kg CO_2_ equivalents/day, respectively; *p* < 0.05).

The higher CF in the IDG dietary pattern was likely due to the significantly higher intake of total whole grains and tubers (0.45 ± 0.08 vs. 0.30 ± 0.06 kg CO_2_ eq./day, respectively) and total dairy foods (0.85 ± 0.43 vs. 0.35 ± 0.07 kg CO_2_ eq./day, respectively), while only a significantly higher intake, and thus CF, of total vegetables was observed in the EAT-IT compared to IDG dietary pattern (0.32 ± 0.13 vs. 0.14 ± 0.06 kg CO_2_ eq./day, respectively) (Figure 2B).

Analyzing the CF in terms of percentage contribution by the different food groups, Figure 2C shows that protein sources were the main contributors in both dietary patterns (36% and 30% in the EAT-IT and IDG-based dietary pattern, respectively). The two dietary patterns were also similar for the % contribution of other food groups such as total whole grains and tubers (11% and 12%, respectively), total fruits (5%), and total oils and seasonings (5% and 7%, respectively). Conversely, they largely differed mainly for dairy foods (13% and 23% in the EAT-IT and IDG-based dietary patterns, respectively) and total sugars (25% and 16%, respectively).

#### 3.1.2. Water Footprint

**Figure 3:**
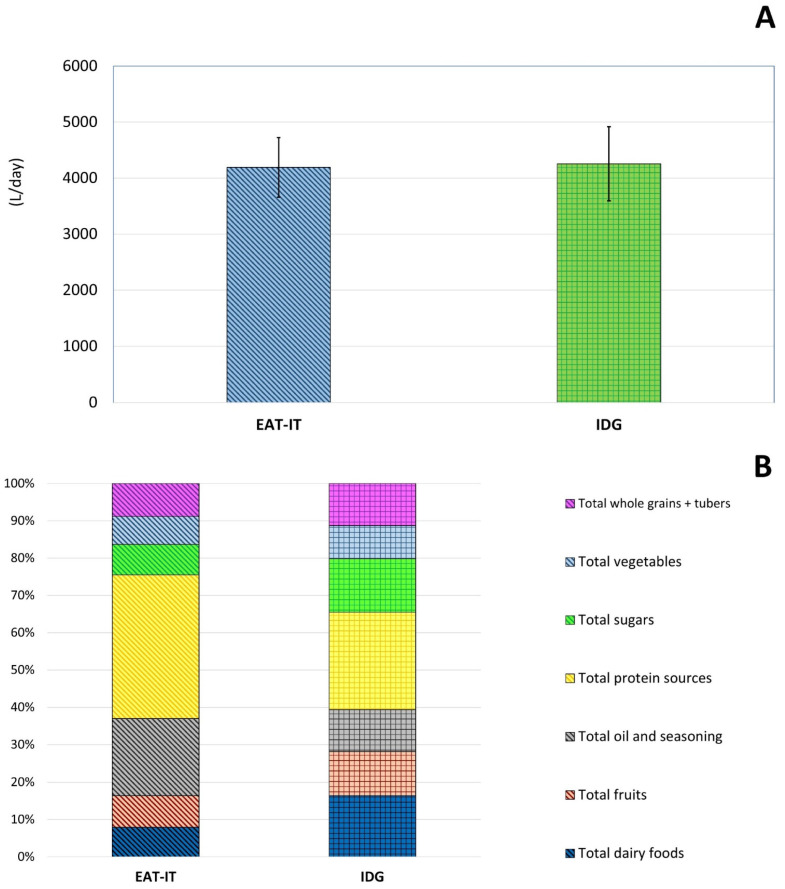

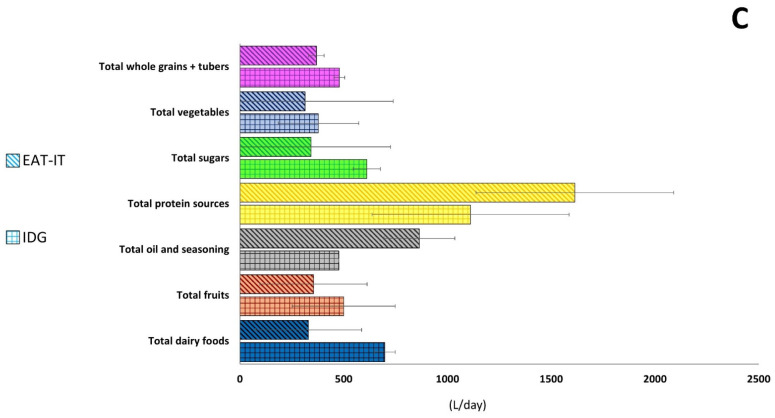
Total (**A**) water footprint associated with EAT-IT and IDG dietary patterns and relative (**B**) and percentage contribution (**C**) of the different food categories. Legend: EAT-IT: EAT-Lancet Commission reference diet (adapted to the Italian food habits); IDG: Italian Dietary Guidelines; WF: water footprint.

Figure 3 shows the WF of the two dietary patterns. WF did not differ between the EAT-IT and IDG-based dietary pattern, being 4189 ± 533 and 4254 ± 662 L/day, respectively (Figure 3A).

Despite the lack of significance between the two dietary patterns, the contribution of the food groups largely varied in the two diets. A higher impact on WF of protein sources and total oils and seasonings was observed in the EAT-IT (Figure 3B), while total fruit and vegetables, dairy foods, and wholegrains and tubers mainly contributed to the impact of the IDG dietary pattern.

As shown in Figure 3C, considering the percentage contribution of the food groups, protein sources were the main contributors in both dietary patterns but varied from 45% in the EAT-IT to 30% in the IDG-based dietary pattern. The dietary patterns also differed for the contribution of total oils and seasonings (23% and 11%, respectively) and total dairy foods (8% and 19 %, respectively), but also total fruits (8% and 12%) and vegetables (4% and 8%).

### 3.2. Analysis of Scenarios Based on Different Food Choices: Case-Studies

**Figure 4:**
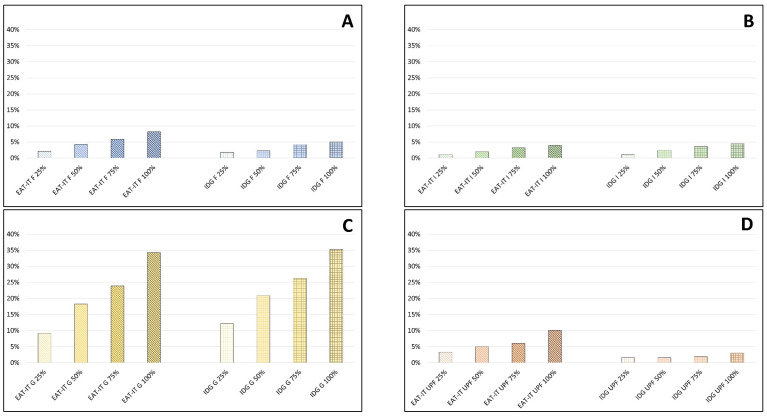
Impact of different food choices on CF and WF case-studies with a gradual increase in the substitution of frozen instead of fresh foods (**A**), imported instead of local fruits (**B**), greenhouse-grown instead of seasonal vegetables (**C**), and processed legume-based foods instead of unprocessed legumes (**D**). Data are expressed as delta percentage compared to the EAT-IT and IDG dietary patterns.

Figure 4 reports the impact of different food choices on CF in four different case-studies. Results show that the gradual increase in the substitution (from 25% to 100%) of frozen instead of fresh foods (Figure 4A), imported instead of local fruits (Figure 4B), greenhouse-grown instead of seasonal vegetables (Figure 4C), and processed legume-based foods instead of unprocessed legumes (Figure 4D) caused an increasing worsening of the CF compared to the related EAT-IT and IDG dietary patterns described in Section 3.1, in which 0% of this substitution was assumed.

In detail, the substitution of frozen instead of fresh foods from 25% to 100% causes an increase in CF from 2.8 to 8.2% and from 1.8 to 5% in EAT-IT and IDG dietary patterns, respectively (Figure 4A), the consumption of imported instead of local fruits from 1.1 to 3.9% and from 1 to 4.5%, while the use of greenhouse-grown instead of seasonal vegetables led to rises from 9 to 34% and from 12 to 39% (Figure 4C). Finally, the consumption of processed legume-based foods (e.g., soy burgers) instead of unprocessed legumes causes an increased CF from 3.4 to 10% and from 1.6 to 3.1% in EAT-IT and IDG dietary patterns, respectively (Figure 4D).

Conversely to CF, WF in the four case-studies was barely affected by these substitutions.

## 4. Discussion

The following study investigated the theoretical carbon and water footprints associated with the EAT-IT dietary pattern, developed based on the planetary diet but adapted to the Italian tradition. The results highlight that this dietary pattern generates a lower CF but not WF compared to a pattern developed based on the IDG. This is not surprising since a recent systematic review that analyzed the environmental outcomes of both empirical and modeling studies on sustainable diets reported an increase in water use (+13.8%) when shifting from baseline to ‘sustainable diets’ [20]. This represents a critical aspect of the definition of healthy and sustainable diets since it indicates that the optimization of one indicator of environmental impact does not automatically correspond to general optimization, and trade-offs are considered likely [21]. Few data are available about the average levels of water footprint of the Italian population. Rosi et al. [22] documented that the food intake of a group of 51 Italian omnivorous adults corresponded to a diet-related water footprint of 3141 L/day. This value differs from those calculated by using our models. In fact, we have found that water footprints for the EAT-IT dietary pattern and IDG were higher compared to the value found by Rosi and colleagues (33.4% and 35.4% for EAT-IT and IDG, respectively); however, data could not be completely compared due to the different databases used for the assessments.

Considering greenhouse gas emissions, the IDG dietary pattern was associated with a higher CF compared to the EAT-IT; however, it is noteworthy that both diets were more favorable compared to the current dietary habits of the Italian population. In fact, according to a recent analysis based on a national food consumption survey, an average CF of 4 kg CO_2_ eq/day was found [23]. Indeed, it has been recently observed that the current consumption of legumes and nuts should be almost doubled to meet the targets proposed by the EAT–Lancet Commission, whereas the consumption of meat, eggs, dairy products, animal fat, tropical oils, and sugars should be largely reduced [24]. The differences observed in CF in the IDG dietary pattern, compared to the EAT-IT one, are probably be due to the higher inclusion of animal-based products (in particular eggs, white meat, milk and derivatives), since they are generally characterized by higher gas emissions compared to plant-based foods [25,26]. These results agree with those by Rosi et al. [22] who found that omnivorous food choices generated higher carbon footprints than vegetarian and vegan diets. Despite these higher footprints in the omnivorous diets, authors found a high inter-individual variability, with a few vegetarian and vegan subjects’ diets showing higher environmental impact than those of some omnivores. Therefore, regardless of the robust and positive effects of plant-based diets on environmental health, results support the role of individual dietary habits in determining the environmental impact of the diet. This is also clearly shown in the present study, in which the large variation (i.e., in terms of standard deviation of CF) within the same food category reflects the effect of single food choices on environmental impact. It should be considered that dietary choice is the result of numerous determinants, and attitudes towards sustainability have been reported to be influenced by gender, location of residence (region and urban vs. non-urban), social class, age, and education [27,28]. For instance, a German nutritional survey on meat consumption carried out in 2019 found that non-meat consumers do not substitute meat with fish, eggs, or milk products but rather with soya products and other plant-based foods. Thus, the evaluation of the magnitude of dietary choices among different food categories or within different food items within the same one is worthy of further study [29]. 

A recent study conducted by Clark and coworkers [30] has estimated for the first time the environmental impact of more than 57,000 food products (including foods belonging to the same food category), commercially available in UK and Ireland. This analysis found that the difference between a most and a least sustainably sourced product (5th and 95th percentile impact, respectively) can be large, depending on ingredient composition [30]. Our work corroborates these findings, since diet-related environmental impacts resulted in significant influences at a food item level, in relation to its production systems and not just the food category to which it belongs [31]. This is further shown by the elaboration of different scenarios, such as the substitution of unprocessed legumes with processed legumes-based foods (e.g., soy burgers), or of local with imported fruits, which can further worsen the environmental impact of the diet, in terms of CF. Interestingly, we found lower exacerbating effects on CF for frozen and imported fruits, reasonably in relation to i) the limited data on frozen products, that did not allow us to find substitute food items in all food categories, ii) the lower weekly amount of total fruits, compared to vegetables. These results support the consumption of seasonal vegetables as a principle of sustainability for plant-based diets, as suggested by the Food and Agriculture Organization of the United Nations (FAO)/ Food Climate Research Network [9]. However, food product seasonality was not included among the 16 guiding principles of sustainable healthy diets proposed in 2019 by FAO/ World Health Organization [32]. Furthermore, a comprehensive review of all available English-translated food-based dietary guidelines indicated that few of them involved environmental sustainability recommendations and the ones that addressed this issue focused on the advice to consume foods produced locally [33]. Given that the inclusion of sustainability criteria within food-based dietary guidelines has been widely advocated [9,10,34,35,36], if our own and previous results are corroborated by other assessments, then a more explicit reference to the seasonality of vegetable products within food-based dietary guidelines could represent a simple and profitable message to be delivered to the population. Another consideration is that despite the four different scenarios which were elaborated separately, their effect on diet-related environmental outcomes can also be additive. The observed increase in final CF when unprocessed legumes were progressively substituted with processed legume-based products was particularly pronounced in the EAT-IT dietary pattern (+0.28 kg of CO_2_ eq./day for 100% substitution), due to the higher presence of legumes in comparison with IDG. However, it should be clear that, according to data provided by the database used, 0.28 kg of CO_2_ eq. corresponds to a portion of just 10.9 g of beef, or 49.0 g of pork. Thus, our analysis aimed to assess the magnitude of food selections among different food items within the same food category on final environmental impact, finding a non-negligible effect on final CF, but largely corroborated the wider literature supporting the lower CF associated with more plant-based diets, in comparison to more meat-based ones [37,38]. The present study has some strengths worth noting. To the best of our knowledge, this is the first study investigating the CF and WF of a dietary model developed based on the planetary diet but adapted to a specific socio-cultural context. Moreover, this study matches perfectly with a previous study in which the nutritional characteristics of the EAT-IT dietary pattern were compared to the IDG one [18], both in their nutritional and environmental aspects, important domains of sustainable healthy diets. However, there are also some limitations. Some of them are strictly related to the use of databases which, although extensive, do not include all food items, and for many of them, provide data related to a high level of uncertainty. Another intrinsic limitation of such databases is the lack of country-specific data reflecting variability across different national food production systems. Moreover, our choice of including only items with both CF and WF values in the database further limited the variety of single items (e.g., types of fruit) included in the model, mainly due to the scarcity of data regarding WF. These observations support the need for improving such databases, increasing and updating their records to allow more precise assessments. Again, in the present study, we investigated the environmental impacts only in terms of CF and WF. However, although these indicators are the two most well-known ones, many other indicators could be used, such as land use and energy use [12]. 

Overall, these results suggest the importance of considering both nutritional and environmental aspects of the diet, with economic and socio-cultural aspects which cannot be neglected to favor the transition towards sustainable healthy diets [39,40]. In this regard, despite the urged necessity to favor the transition towards sustainable food systems, literature supports the importance of considering social–cultural aspects and country-specific food habits, since a gradual transition to a sustainable diet is likely to be more easily implementable by the population, and is likely to arise from a general shift towards a healthier lifestyle and social environment [29,30,31,41,42]. To this aim, further evaluations, including aspects related to diet affordability and acceptability, will be crucial for a better comprehension of the real applicability of these dietary sustainable models. However, considering data already present in literature, it is plausible to estimate that the shift to a more sustainable healthy diet such as the EAT-IT diet would result in lower costs and thus would be more affordable than the less sustainable diets, especially in the upper-middle- and high-income countries [43]. 

The importance of a holistic approach to contextualize the environmental impacts of different diets has recently been advocated [44]. For instance, given the different bioavailability of compounds in plant-based foods and animal-source foods, it has been recommended that future research should give more consideration to the inclusion of bioavailability assessment within nutrition metrics. This will be important to predict and understand the potential positive/negative impact of a major or minor bioavailability of a specific nutrient/non-nutrient on human health (in particular when considering vulnerable groups of populations), which in turn allows better comprehension of the different dimensions of sustainability [44,45,46,47]. In this scenario, Ferrari et al. [23] tried to optimize a healthy and sustainable dietary model with low gas emission, satisfying dietary requirements and taking into consideration current Italian food consumption patterns, but they could not find any suitable diet fulfilling the iron constraint for females, which remained below the dietary reference intake in the optimized model, supporting the importance of a holistic approach when developing these dietary models.

## 5. Conclusions

In conclusion, our analysis integrates the previous evaluation of the nutritional adequacy of a plant-based dietary pattern based on the Planetary diet and adapted to the Italian-Mediterranean food context (EAT-IT dietary pattern). Our results demonstrated a lower CF of this pattern compared to an IDG one, but not reduced levels of WF, thus supporting the concept that optimization of one indicator of environmental impact does not necessarily allow the optimization of all other indicators. Furthermore, we found that individual food choices can markedly influence final diet-related environmental outcomes, showing that limiting animal food products may not be enough to achieve the goal of a low CF-diet. In particular, the worst scenario occurred when large shares of vegetables were substituted with vegetables grown in heated greenhouses and when legumes were substituted with processed legume-based foods, such as soy burgers. Eating fruit and vegetables according to seasonality can represent a viable message to be included in sustainable diet recommendations. Overall, our analysis corroborates the wide scientific literature reporting the lower CF associated with plant-based diets, in comparison with the ones based on a higher consumption of animal foods. Finally, a holistic approach to the evaluation of environmentally optimized diets should be carefully considered to safeguard all the dimensions of a sustainable healthy diet. 

## Data Availability

The data that support the findings of this study are included in the paper.

## References

[1] Afshin A., Sur P.J., Fay K.A., Cornaby L., Ferrara G., Salama J.S., Mullany E.C., Abate K.H., Abbafati C., Abebe Z. (2019). Health effects of dietary risks in 195 countries, 1990–2017: A systematic analysis for the Global Burden of Disease Study 2017. Lancet.

[2] Crippa M., Solazzo E., Guizzardi D., Monforti-Ferrario F., Tubiello F.N., Leip A. (2021). Food systems are responsible for a third of global anthropogenic GHG emissions. Nat. Food.

[3] Mrówczyńska-Kamińska A., Bajan B., Pawłowski K.P., Genstwa N., Zmyślona J. (2021). Greenhouse gas emissions intensity of food production systems and its determinants. PLoS ONE.

[4] Food and Agriculture Organization of the United Nation (2021). The State of the World’s Land and Water Resources for Food and Agriculture–Systems at Breaking Point (SOLAW 2021).

[5] Whitmee S., Haines A., Beyrer C., Boltz F., Capon A.G., Ferreira B., Dias D.S., Ezeh A., Frumkin H., Gong P. (2015). The Rockefeller Foundation—Lancet commission on planetary health Safeguarding human health in the Anthropocene epoch: Report of the Rockefeller Foundation—Lancet commission on planetary health. Lancet.

[6] Fanzo J., Davis C. (2019). Can diets be healthy, sustainable, and equitable?. Curr. Obes. Rep..

[7] Fanzo J., McLaren R., Davis C., Choufani J. (2017). Climate Change and Variability: What Are the Risks for Nutrition, Diets, and Food Systems?. IFPRI Discussion Paper 1645.

[8] Burlingame B., Dernini S., Food and Agriculture Organization of the United Nations, Bioversity International (2012). Sustainable Diets and Biodiversity.

[9] Fischer C.G., Garnett T. (2016). Plates, Pyramids, Planet.

[10] Springmann M., Wiebe K., Mason-D’Croz D., Sulser T.B., Rayner M., Scarborough P. (2018). Health and nutritional aspects of sustainable diet strategies and their association with environmental impacts: A global modelling analysis with country-level detail. Lancet Planet. Health.

[11] Willett W., Rockström J., Loken B., Springmann M., Lang T., Vermeulen S., Garnett T., Tilman D., DeClerck F., Wood A. (2019). Food in the anthropocene: The EAT—Lancet commission on healthy diets from sustainable food systems. Lancet.

[12] van Dooren C., Aiking H., Vellinga P. (2018). In search of indicators to assess the environmental impact of diets. Int. J. Life Cycle Assess..

[13] Petersson T., Secondi L., Magnani A., Antonelli M., Dembska K., Valentini R., Varotto A., Castaldi S. (2021). A multilevel carbon and water footprint dataset of food commodities. Sci. Data.

[14] Rockström J., Stordalen G.A., Horton R. (2016). Acting in the anthropocene: The EAT—Lancet commission. Lancet.

[15] Lassen A.D., Christensen L.M., Trolle E. (2020). Development of a Danish adapted healthy plant-based diet based on the EAT—Lancet reference diet. Nutrients.

[16] Sharma M., Kishore A., Roy D., Joshi K. (2020). A comparison of the Indian diet with the EAT—Lancet reference diet. BMC Public Health.

[17] Blackstone N.T., Conrad Z. (2020). Comparing the recommended eating patterns of the EAT—Lancet commission and dietary guidelines for Americans: Implications for sustainable nutrition. Curr. Dev. Nutr..

[18] Tucci M., Martini D., Del Bo’ C., Marino M., Battezzati A., Bertoli S., Porrini M., Riso P. (2021). An Italian-Mediterranean dietary pattern developed based on the EAT—Lancet Reference Diet (EAT-IT): A nutritional evaluation. Foods.

[19] Leclercq C., Arcella D., Piccinelli R., Sette S., Le Donne C., Turrini A., INRAN-SCAI 2005–06 Study Group (2009). The Italian National Food Consumption Survey INRAN-SCAI 2005–06: Main results in terms of food consumption. Public Health Nutr..

[20] Jarmul S., Dangour A.D., Green R., Liew Z., Haines A., Scheelbeek P.F.D. (2019). Climate change mitigation through dietary change: A systematic review of empirical and modelling studies on the environmental footprints and health effects of “sustainable diets”. Environ. Res. Lett..

[21] Garnett T. (2014). What is a Sustainable Healthy Diet?. A Discussion Paper.

[22] Rosi A., Mena P., Pellegrini N., Turroni S., Neviani E., Ferrocino I., Di Cagno R., Ruini L., Ciati R., Angelino D. (2017). Environmental impact of omnivorous, ovo-lacto-vegetarian, and vegan diet. Sci. Rep..

[23] Ferrari M., Benvenuti L., Rossi L., De Santis A., Sette S., Martone D., Piccinelli R., Le Donne C., Leclercq C., Turrini A. (2020). Could dietary goals and climate change mitigation be achieved through optimized diet? The experience of modeling the national food consumption data in Italy. Front. Nutr..

[24] Vitale M., Giosuè A., Vaccaro O., Riccardi G. (2021). Recent trends in dietary habits of the italian population: Potential impact on health and the environment. Nutrients.

[25] Clark M.A., Springmann M., Hill J., Tilman D. (2019). Multiple health and environmental impacts of foods. Proc. Natl. Acad. Sci. USA.

[26] Poore J., Nemecek T. (2018). Reducing food’s environmental impacts through producers and consumers. Science.

[27] Gossard M.H., York R. (2003). Social structural influences on meat consumption. Hum. Ecol. Rev..

[28] Kirbiš A., Lamot M., Javornik M. (2021). The role of education in sustainable dietary patterns in Slovenia. Sustainability.

[29] Koch F., Heuer T., Krems C., Claupein E. (2019). Meat consumers and non-meat consumers in Germany: A characterisation based on results of the German National Nutrition Survey II. J. Nutr. Sci..

[30] Clark M., Springmann M., Rayner M., Scarborough P., Hill J., Tilman D., Macdiarmid J.I., Fanzo J., Bandy L., Harrington R.A. (2022). Estimating the environmental impacts of 57,000 food products. Proc. Natl. Acad. Sci. USA.

[31] Kim B.F., Santo R.E., Scatterday A.P., Fry J.P., Synk C.M., Cebron S.R., Mekonnen M.M., Hoekstra A.Y., de Pee S., Bloem M.W. (2020). Country-specific dietary shifts to mitigate climate and water crises. Glob. Environ. Chang..

[32] FAO, WHO (2019). Sustainable Healthy Diets–Guiding Principles.

[33] Martini D., Tucci M., Bradfield J., Di Giorgio A., Marino M., Del Bo’ C., Porrini M., Riso P. (2021). Principles of sustainable healthy diets in worldwide dietary guidelines: Efforts so far and future perspectives. Nutrients.

[34] Springmann M., Spajic L., Clark M.A., Poore J., Herforth A., Webb P., Rayner M., Scarborough P. (2020). The healthiness and sustainability of national and global food based dietary guidelines: Modelling study. BMJ.

[35] Bechthold A., Boeing H., Tetens I., Schwingshackl L., Nöthlings U. (2018). Perspective: Food-based dietary guidelines in Europe—Scientific concepts, current status, and perspectives. Adv. Nutr..

[36] Magni P., Bier D.M., Pecorelli S., Agostoni C., Astrup A., Brighenti F., Cook R., Folco E., Fontana L., Gibson R.A. (2017). Perspective: Improving nutritional guidelines for sustainable health policies: Current status and perspectives. Adv. Nutr..

[37] Tilman D., Clark M. (2014). Global diets link environmental sustainability and human health. Nature.

[38] Chai B.C., van der Voort J.R., Grofelnik K., Eliasdottir H.G., Klöss I., Perez-Cueto F.J.A. (2019). Which diet has the least environmental impact on our planet? A systematic review of vegan, vegetarian and omnivorous diets. Sustainability.

[39] Perignon M., Masset G., Ferrari G., Barré T., Vieux F., Maillot M., Amiot M., Darmon N. (2016). How low can dietary greenhouse gas emissions be reduced without impairing nutritional adequacy, affordability and acceptability of the diet? A modelling study to guide sustainable food choices. Public Health Nutr..

[40] FAO, IFAD, UNICEF, WFP, WHO (2020). In Brief to The State of Food Security and Nutrition in the World 2021. Transforming Food Systems for Food Security, Improved Nutrition and Affordable Healthy Diets for All.

[41] Collins A., Fairchild R. (2007). Sustainable food consumption at a sub- national level : An ecological footprint, nutritional and economic analysis. J. Environ. Policy Plan..

[42] Downs S.M., Ahmed S., Fanzo J., Herforth A. (2020). Food environment typology: Advancing an expanded definition, framework, and methodological approach for improved characterization of wild, cultivated, and built food environments toward sustainable diets. Foods.

[43] Springmann M., Clark M.A., Rayner M., Scarborough P., Webb P. (2021). The global and regional costs of healthy and sustainable dietary patterns: A modelling study. Lancet. Planet. Health.

[44] Dave L.A., Hodgkinson S.M., Roy N.C., Smith N.W., McNabb W.C. (2021). The role of holistic nutritional properties of diets in the assessment of food system and dietary sustainability. Crit. Rev. Food Sci. Nutr..

[45] HLPE (2020). Food Security and Nutrition: Building a Global Narrative towards 2030. A Report by the High Level Panel of Experts on Food Security and Nutrition of the Committee on World Food Security.

[46] Smith N.W., Fletcher A.J., Hill J.P., McNabb W.C. (2022). Modeling the contribution of meat to global nutrient availability. Front. Nutr..

[47] Leroy F., Abraini F., Beal T., Dominguez-Salas P., Gregorini P., Manzano P., Rowntree J., van Vliet S. (2022). Animal board invited review: Animal source foods in healthy, sustainable, and ethical diets—An argument against drastic limitation of livestock in the food system. Animal.

